# COVID-19 Conversion after Exposure in a Semiprivate Room at a Tertiary Care Center in Iowa, July–December 2020

**DOI:** 10.1017/ash.2021.37

**Published:** 2021-07-29

**Authors:** Alexandra Trannel, Takaaki Kobayashi, Oluchi Abosi, Kyle Jenn, Holly Meacham, Lorinda Sheeler, William Etienne, Angie Dains, Mary Kukla, Mohammed Alsuhaibani, Stephanie Holley, Alexandre Marra, Bradley Ford, Melanie Wellington, Daniel Diekema, Jorge Salinas

## Abstract

**Background:** Hospital semiprivate rooms may lead to coronavirus disease 2019 (COVID-19) patient exposures. We investigated the risk of COVID-19 patient-to-patient exposure in semiprivate rooms and the subsequent risk of acquiring COVID-19. **Methods:** The University of Iowa Hospitals & Clinics is an 811-bed tertiary care center. Overall, 16% of patient days are spent in semiprivate rooms. Most patients do not wear masks while in semiprivate rooms. Active COVID-19 surveillance included admission and every 5 days nasopharyngeal SARS-CoV-2 polymerase chain reaction (PCR) testing. We identified inpatients with COVID-19 who were in semiprivate rooms during their infectious periods during July–December 2020. Testing was repeated 24 hours after the first positive test. Cycle threshold (Ct) values of the two tests (average Ct <30), SARS-CoV-2 serology results, clinical assessment, and COVID-19 history were used to determine patient infectiousness. Roommates were considered exposed if in the same semiprivate room with an infectious patient. Exposed patients were notified, quarantined (private room), and follow-up testing was arranged (median seven days). Conversion was defined as having a negative test followed by a subsequent positive within 14 days after exposure. We calculated the risk of exposure: number of infectious patients in semiprivate rooms/number of semiprivate patient-days (hospitalization days in semiprivate rooms). **Results:** There were 16,427 semiprivate patient days during July–December 2020. We identified 43 COVID-19 inpatients who roommates during their infectious periods. Most infectious patients (77%) were male; the median age was 67 years; and 22 (51%) were symptomatic. Most were detected during active surveillance: admission testing (51%) and serial testing (28%). There were 57 exposed roommates. The risk of exposure was 3 of 1,000 semiprivate patient days. In total, 16 roommates (28%) did not complete follow-up testing. Of 41 exposed patients with follow-up data, 8 (20%) converted following their exposure. Median time to conversion was 5 days. The risk of exposure and subsequent conversion was 0.7 of 1,000 semiprivate patient days. Median Ct value of the source patient was 20 for those who converted and 23 for those who did not convert. Median exposure time was 45 hours (range, 3–73) for those who converted and 12 hours (range, 1–75) for those who did not convert. **Conclusions:** The overall risk of exposure in semiprivate rooms was low. The conversion rate was comparable to that reported for household exposures. Lower Ct values and lengthier exposures may be associated with conversion. Active COVID-19 surveillance helps early detection and decreases exposure time.

**Funding:** No

**Disclosures:** None

